# Food beliefs and practices in urban poor communities in Accra: implications for health interventions

**DOI:** 10.1186/s12889-018-5336-6

**Published:** 2018-04-02

**Authors:** Sandra Boatemaa, Delali Margaret Badasu, Ama de-Graft Aikins

**Affiliations:** Regional Institute for Population Studies, University of Ghana, Accra-Legon, Ghana

**Keywords:** Food beliefs, Healthy eating, Unhealthy eating, Socio-ecological model, Ghana

## Abstract

**Background:**

Poor communities in low and middle income countries are reported to experience a higher burden of chronic non-communicable diseases (NCDs) and nutrition-related NCDs. Interventions that build on lay perspectives of risk are recommended. The objective of this study was to examine lay understanding of healthy and unhealthy food practices, factors that influence food choices and the implications for developing population health interventions in three urban poor communities in Accra, Ghana.

**Methods:**

Thirty lay adults were recruited and interviewed in two poor urban communities in Accra. The interviews were audio-taped, transcribed and analysed thematically. The analysis was guided by the socio-ecological model which focuses on the intrapersonal, interpersonal, community, structural and policy levels of social organisation.

**Results:**

Food was perceived as an edible natural resource, and healthy in its raw state. A food item retained its natural, healthy properties or became unhealthy depending on how it was prepared (e.g. frying vs boiling) and consumed (e.g. early or late in the day). These food beliefs reflected broader social food norms in the community and incorporated ideas aligned with standard expert dietary guidelines. Healthy cooking was perceived as the ability to select good ingredients, use appropriate cooking methods, and maintain food hygiene. Healthy eating was defined in three ways: 1) eating the right meals; 2) eating the right quantity; and 3) eating at the right time. Factors that influenced food choice included finances, physical and psychological state, significant others and community resources.

**Conclusions:**

The findings suggest that beliefs about healthy and unhealthy food practices are rooted in multi-level factors, including individual experience, family dynamics and community factors. The factors influencing food choices are also multilevel. The implications of the findings for the design and content of dietary and health interventions are discussed.

**Electronic supplementary material:**

The online version of this article (10.1186/s12889-018-5336-6) contains supplementary material, which is available to authorized users.

## Background

Poor diets (meals high in salt, fats and sugar) are associated with the rising levels of obesity and chronic non-communicable diseases globally [1]. Sub-Saharan Africa has a growing burden of obesity and NCDs and research attributes this burden, partly to poor diets and physical inactivity [2–5]. Between 1980 and 2009, per capita calorie and sugar supply increased to levels greater than the recommended levels in fifteen and seventeen Saharan African countries (SSA), respectively [6].

The NCD burden in SSA and low and middle income countries (LMICs) is stratified along socio-economic lines. Researchers observe a protracted polarised model of the epidemiological transition, where the wealthy experience high NCD risk, and the poor experience a double jeopardy of NCDs and infectious diseases [2, 7]. Demographic and health surveys and other data sources indicate that obesity among women in urban areas is increasing, and especially among the poor [8–11]. In urban poor communities about a third of women are overweight or obese and majority of these women have undernourished children [12].

The food environment of urban poor communities is obesogenic and characterised by low cost, high energy dense street foods, processed foods and fast foods [5, 13–15]. Poor households, and groups with low education, are less likely to consume fruit and vegetables [16–18].

Research examining food habits in Africa suggests that communities do not adhere to expert dietary guidelines due to cultural food beliefs and habits [19–22]. In Ghana, the high intake of fat, sugar and salt and low intake of fruits and vegetables have been reported to arise from structural, material and symbolic barriers [23]. More than half of the urban Ghanaian population (58%) lives in poor communities [24]. Prevalence rates of obesity, hypertension, diabetes and other NCDs are rising in these communities [13, 25, 26]. However, healthcare for NCDs is not accessible or equitable in these communities [25, 27]. In addition, research examining the socio-cultural context of food beliefs and practices and of nutrition-related NCDs in urban poor Ghanaian communities is limited [13].

It has been suggested that health interventions that build on rather than challenge existing health beliefs can improve health behaviour [28, 29]. Understanding lay perspectives on food, food habits and behaviour can inform primary and secondary obesity and NCDs intervention strategies [30, 31]. It is critical to understand and address the impact of diet on NCDs, especially in countries and communities disproportionately affected by NCDs [1]. As part of a large-scale qualitative study of cardiovascular disease (CVD) prevention and management – aspects of which are reported elsewhere [25–27] - we examined the beliefs of healthy and unhealthy food practices in three urban poor communities in Accra: Agbobloshie, James Town and Ussher Town. This paper focuses on three areas of enquiry: food beliefs; beliefs about healthy and unhealthy food practices; and factors that influence food choice. These areas provide preliminary information for developing primary and secondary NCD interventions for the research communities.

### Conceptual framework

Food beliefs incorporate individual and societal ideals about food. They reflect social and culturally acquired knowledge on food, carefully selected and maintained over time and are essential determinants of food behaviour [30, 32]. Research on food beliefs and behaviours has been influenced by sociological, psychological, anthropological and public health models [33]. It has been suggested that residents in a community encounter, modify and adopt the dietary beliefs of their communities [28, 32]. In the urban food environment a mixture of traditional and contemporary food beliefs co-exist [34].

The socio-ecological model proposed by McLeroy and colleagues [35] outlines five levels of influence on behaviour: intra and interpersonal factors, community and structural factors (or institutional) and public policy. They argue for a reciprocal causation where behaviour both affects and is affected by multiple levels of the social environment [34]. The socio-ecological model has been used extensively to determine factors that influence food beliefs and practices and health outcomes [34, 36, 37]. In general, the socio-ecological perspective provides an understanding of individual and environmental causes of behaviour as well as insights for developing context-specific interventions [35]. The health challenges of the study community are shaped by social-cultural and environmental factors. Therefore understanding food beliefs, habits and NCD risk from a socio-ecological perspective is an appropriate approach.

The Ministry of Health (MOH) of Ghana implemented the Regenerative Health and Nutrition Programme (RHNP) in 2007 to promote healthy lifestyle and reduce the risk of NCDs [20, 28, 29]. A review of the program identified multi-level barriers to adoption and practice of healthy lifestyles. These included individual factors such as taste and limited personal finances, and sociocultural factors such as the legitimation of culturally accepted diets and stigmatization of diet preferences such as vegetarianism [38, 39].

In this paper, we used the socio-ecological model to understand what drives food beliefs and practices. We conceptualised the five levels of influence as follows. At the intrapersonal level food choices could be informed by an individual’s knowledge of different foods, preferences, taste and psychological state. At the interpersonal level the patterns of household food preparation and food habits of significant others could inform the food choices of individuals [40]. At the community level, food availability and prices could influence the accessibility of food and food choices [12, 41]. At the structural and public policy levels, food habits and choices could be shaped by broader community food beliefs and norms, food regulations and policy [41].

## Methods

### The study communities

Accra, like many SSA countries, is undergoing rapid urbanisation, which is attributed largely to rural-urban migration [42]. Urban communities are classified as rich or poor based on their access to social amenities such as water, housing, electricity, improved toilet and population density [43–46]. In 2002 Zulu et al. [45] developed a proxy measure for slum communities, where communities that lacked running water, electricity and improved toilet facilities were considered as slum [45]. Other researchers consider informal settlements as slums [47, 48]. In Accra, Agbogloshie – one of our study communities – is an example of a 205 slum settlements [49]. Some formal settlements that lack social amenities are considered as urban poor communities. One such example is our other study community, Ga Mashie.

Agbobloshie, James Town and Ussher Town (the last two communities are twinned and referred to as Ga Mashie) are formally labelled urban poor by the Accra Metropolitan Assembly (AMA) because of their low socio-economic status compared to the national average. Ga Mashie is a densely populated community. The residents have low levels of education and live in congested housing [24]. Ga Mashie is mainly inhabited by the indigenous Ga-Dangme ethnic group. About 50 % of the residents work in the food industry as street food vendors, fishmongers, food processors and/or bakers [50]. Agbobloshie is also an unplanned neighbourhood occupied mostly by squatters and rural urban migrants in temporary wooden structures. Agbobloshie has a heterogeneous ethnic composition as a result of in-migration; most of the residents are traders or artisans in the five markets that surround the community.

### Data collection

This qualitative study is part of a broader longitudinal study of population, environment and health in Agbobloshie, James Town and Ussher Town by the Regional Institute for Population Studies (RIPS). RIPS, in partnership with the Secretariat of the African Caribbean and Pacific Group of States – ACP-EU Cooperation Programme in Higher Education (EDULINK) and the International Development Research Centre (IDRC), has established an active research field site in the three communities. The longitudinal study of Urban Poverty and Health Survey aims to examine health, cardiovascular diseases, poverty and development indicators in these settings over time and to provide vital data to local and regional stakeholders for the development of the communities. Three rounds of survey with households and individuals were conducted at 18–20 month intervals in 2010 (June), 2011 (December) and 2013 (September), with households and individuals.

A mixed qualitative study was conducted as part of the Round 2 of the Urban Poverty and Health Survey and was part of a broader longitudinal CVD study reported elsewhere [25, 27].

The qualitative study examined beliefs, knowledge, habits and experiences of NCDs, as well as representations of risk factors, including diet, overweight and obesity. This paper focuses on individual data on food beliefs and practices. The population of interest for this study were residents of the study area aged 15 years and above. To ensure sample heterogeneity residents were selected from the age groups 15–35 and 35+ by sex and locality. Participants from the survey were visited by the first author and asked whether they were interested in participating in a study on food beliefs and practices. Appointments were made with interested persons. Fifteen of the interviewees were participants of the EDULINK survey, while 15 interviewees did not participate in the survey.

An interview guide, structured into four sections was used for the interviews: personal history of participants; lay beliefs of healthy and unhealthy food practices; beliefs about cooking oil and salt; and dietary practices. Table 1 summarises the interview themes and probes. Semi structured interviews were conducted from January 2012 to June 2012, by three trained interviewees, with thirty participants at locations of participants’ choice. The interviews were conducted in English (*n* = 4), Twi (*n* = 25) and Ga (*n* = 1). Interviews lasted between 40 and 90 min. The interview guide served as a start to the discussion, but particular attention was also paid to ideas shared by the participants for further elaboration. Notes as well as audio tapes were taken for all interviews.

**Table 1:** Sample questions from the interview guide

Section	Main question	Probes
Lay food beliefs	What is food in general?	How would you define food? What would you consider as food?
	Are there meanings attached to foods?	How do people get to decide on what to eat/not to eat?
	Do you have meanings attached to the food you eat?	Do people eat certain foods to portray an identity (e.g. Wealth, religious meals, well-being, celebrations and ethnicity)?
(Un)Healthy eating	What would you consider to be good/bad food?	List ten of such?
	What would you consider to be (un)healthy eating?	Time of eating?
		Does how much food a person eat matters?
		What about the content of food (Ingredients/lack/ low intake of vegetables and fruits)?
		Does the source (Home, street vendor) of the food matter?
	What factors influence your food choice?	Individual (Finances, health status)
		Family (Spouse, children, other relatives and friends),
		Community (Food availability, food prices)
		Structural (Government laws, poverty)

### Data analysis

The analysis was conducted after all the interviews had been conducted, transcribed and quality checked. The audio-tape of the English and Twi interviews were transcribed verbatim into English by SB and the Ga interviews by a trained research assistant. The RIPS research team has cultural and multi-lingual competence having worked in the community for a number of years. A designated language quality checker (a PhD candidate at RIPS) with English, Ga and Twi skills was asked to listen to the audio files alongside the transcripts. Secondly, the qualitative research team also applied best practices – such as developing a linguistic diary, discussing language issues and reconciling conflicting narratives.

The data were analysed using thematic analysis as outlined by Attride-Stirling [51]. The analysis covered personal history of the participants and lay beliefs of healthy and unhealthy food practices. The analysis was started by reading transcripts to understand the data and identify emerging codes and themes. The analytical framework was guided by both deductive and inductive codes. The *deductive codes* were derived from previous qualitative food studies conducted in Ghana [25, 52–54]. The *inductive codes* were anticipated from the culturally specific contexts of the three communities. Previous studies in Ghana showed that perceptions of healthy eating included high intake of fruits, vegetables, early meals, boiling and food hygiene (Table 2). In developing the coding frame these reported ideas from previous research were identified as deductive codes. The coding process was started by identifying these codes.

**Table 2:** Established perceptions of healthy and unhealthy eating and factors that influence food habits

Perceptions of healthy eating	Perceptions of unhealthy eating	Factors that influence food habits
Cooking habits (boiling, roasting, food hygiene)	Cooking habits (frying)	Antenatal and Post-natal food culture
Food intake (high fruit and vegetable intake, early meals)	Eating habits (high intake of fatty foods, sugar, refined carbohydrates, late meals)	Poverty
Meal content (use of fresh vegetables)		Significant others (Spouse, children)
		Religion (forbidden versus approved foods)
		Ethnicity (ethnic foods, food taboos)

Source: 7,17,20,22,56

**Fig. 1:**
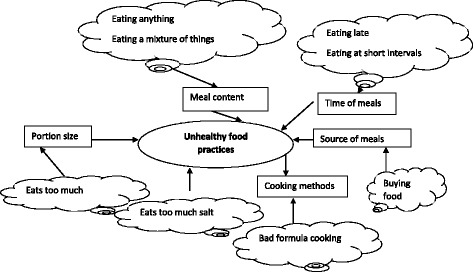
A diagram to illustrate the coding process from basic to organising to global theme.

The second stage of the analysis involved identifying the linkages between codes, themes, and appropriate respondent quotes, existing research and the conceptual framework. Figure 1 displays the relationships between basic, organising and global themes for perceptions of unhealthy food practices. For example, codes such as ‘eating anything’ and ‘eating a mixture of things’ was interpreted as defining unhealthy eating in relation to meal content.

### The participants

The characteristics of respondents are presented in summary and in detail in Table 3 and Additional file 1, respectively. Seventeen women and thirteen men aged between 15 and 67 years were interviewed. The interviewees were of different marital status (never married, currently married and formerly married). Thirteen participants resided at James Town, nine in Ussher Town and eight at Agbobloshie. All the participants had formal education with the exception of three who had never been to school. The participants were from different ethnic backgrounds (Ga-Dangme, Akan, Ewe and Mole-Dagbani) and were mostly Christians (*n* = 28). One woman self-reported living with hypertension. Half of the participants were traders.

**Table 3:** Characteristics of interviewees

Variable	Percent	Number
Age		
< 20	13.3	4
21–30	26.7	8
31–40	26.7	8
41–50	13.3	4
50+	20.0	6
Sex		
Male	43.3	13
Female	56.7	17
Ethnicity		
Akan	30.0	9
Ga-Dangme	63.4	19
Ewe	3.3	1
Mole-Dagbani	3.3	1
Education		
None	10.0	3
Primary	16.7	5
Middle/JSS	33.3	10
Secondary	33.3	10
Higher	6.7	2
Locality		
Agbobloshie	26.7	8
James Town	43.3	13
Ussher Town	30.0	9
Religion		
Christian	93.3	28
Moslem	2.7	2
Health profile		
Do not live with hypertension	96.7	29
Lives with hypertension	3.3	1
Occupation		
Unemployed/student/Retired	16.7	5
Trader	50	15
Mason	3.3	1
Hair/dress stylist	20	6
Printer/Civil Servant/ Security Man	10	3
Total	100.0	30

## Results

The findings of the study are grouped under four themes: (1) food beliefs (2) beliefs of healthy and unhealthy cooking habits (3) beliefs of healthy and unhealthy eating and (4) factors that influence food choice. We include the spread of views by indicating the number of participants that shared a food belief in parentheses.

### Food beliefs

The participants considered food as any natural resource that can be eaten (*n* = 9) and is socially accepted as food (*n* = 2). Close to half of the participants mentioned that foods were supposed to provide nutrients to aid in growth, health, strength and support life. The absence of food in human life was associated with the loss of vital nutrients and bodyweight. About a third of participants believed that food in natural/r aw state was healthy, regardless of its regional and/or ethnic origin. According to some participants, what made a food healthy or unhealthy to the body was the cooking method (*n* = 5) and mode of consumption (*n* = 6).

*‘All food is good, it depends on the time you eat it that will or won’t make you have your peace of mind.’* (R25).

*‘You see, God is wonderful, you see God provide everything that human beings need. So everything is good, every food is good, but too much of everything is bad. Poor cooking makes food unhealthy.*’ (R4).

Some healthy foods mentioned were *fufu, banku,* boiled rice*, kenkey,* oats, *smoked fish, dry smoked fish,* oats*, ampesi*, *palava sauce,* cabbage stew, fruits and vegetables [carrot, cucumber] (Table 4) (see Additional file 2 for description of food items). Healthy foods were believed to have blood-giving properties. Examples of unhealthy foods mentioned were *oily foods*, fried rice, fried pork, *Indomie instant noodle*s [a popular brand of instant noodles], fried egg, sausage and beer. The unhealthy foods were perceived to cause diseases and disturbance to the body.

**Table 4:** Examples of healthy and unhealthy foods mentioned by participants

Food group	Healthy foods	Unhealthy foods	Reason for classification
Tubers and plantains	*Fufu*, *ampesi, kokonte*		
Cereals	*Banku*, boiled rice, *kenkey*, oats	Fried rice, *oiled rice*, indomie instant noodles	Oiled rice gives fever
Fat and oils	Palm oil	*toogbe*, *kelewele*, fried foods, *oily foods (“foods you can squeeze oil out of*”)	
Vegetables	Palm nut soup, light soup, *palava sauce*, cabbage stew, cucumber, cocoyam leaves, carrot		*Palava sauce* gives *“blood”* (stew made with cocoyam leaves, melon seeds and fish)
			Vegetables gives strength and long life
Animal foods	Smoked fish, dry smoked fish, *akola*	Sausage, meat, khebab, fried egg, fried pork, chicken skin	
Fruits	Mango, banana		
Legumes	Beans		
Alcohol		Beer	
Other			
Dry foods		Dry food or unbalanced foods	Dry foods (made with less vegetables and protein ingredients but with a lot of carbohydrates)
Toxic foods		Foods planted with fertilizer	*“Those chemicals disturbs our system”*

### Beliefs of healthy and unhealthy cooking habits

Healthy cooking was perceived by respondents in three ways: healthy food preparation; food content; and food hygiene.

#### Healthy food preparation

Healthy food preparation was described by respondents as choosing the appropriate cooking methods and applying it well. Boiling, roasting and stewing was seen as healthy cooking methods while frying was considered unhealthy. It was suggested that when frying food should be fried in hot oil to avoid the food soaking too much oil. Also, healthy food preparation meant not overcooking or undercooking a meal.

‘*It depends on how you cook it. You fry tatale* [fried ripe plantain fritters] *in oil that is not hot and it soaks the oil, that is not good.’* (R25).

*‘Unhygienic food and half cooked food you see now, when you cook the food half way it is not good.’* (R5).

#### Healthy food content

The content of food was used by respondents to describe healthy cooking. A healthy meal was made from fresh, uncontaminated raw ingredients and those without toxic fertiliser residue. Ingredients without blemish were the ideal ingredients for meal preparation. Healthy foods were also described as those which contained less artificial spices. Nearly half of the respondents considered healthy foods as those that contained all nutrients; foods that had *‘not too much oil,*’ (R1, R4, R15, and R24), ‘*not too much starch,*’ (R16, and R19) but also other nutrients (*n* = 15).

*‘We have some foods that require oil. But there are others too that do not require oil. So the ones you are supposed to use oil, you use it, the ones you are not supposed to, you don’t. Yes, when you eat too much of it, when you eat too much it can give you problems in your body. When you are frying chicken and you use too much oil when you squeeze it you will see that the oil is coming out and when you use it [oil] to cook oil rice after serving you will find plenty oil under the saucepan.’* (R15).

*‘Maggi* [a popular artificial seasoning] *sometimes, me as for maggi and those things I don’t like them. Because some of the maggi they use it for polishing the cooking utensils, how can they use maggi to cook and at the same time use it to polish utensils, it is bad cooking.’* (R6).

Participants also mentioned that healthy meals had to be tasty and delicious (*n* = 6). Some participants expressed worry about the tastelessness of some healthy foods (*banku* with less salt) and avoided such food. They emphasised that some unhealthy foods such as instant noodles and fried rice have become popular foods because of their delicious taste.

*‘In the afternoon I ate indomie. I saw someone eating it and it attracted me. The TeleNurse said it is bad for health, but it is nice.’ (R26).*

#### Food hygiene

Food hygiene was mentioned as a healthy food practice. According to respondents, food had to be cooked and served in a clean environment and with clean equipment. The environment had to be free of flies, gutters, rubbish and dust. Inadequate cleaning of kitchen utensils and equipment and poor personal hygiene during food preparation was perceived to generate germs, bacteria and contaminants that are harmful to the body. Seven of the participants were concerned about the food hygiene of vendor foods.

*‘Ok, where they cook the food and the way those who sell the food look like that is how you would know whether the food you are going to eat is good or bad. Someone could be selling rice just here another person too, maybe selling the rice as well, but I buy from the one who is neat. Someone might cook here another person might also be cooking here, but when you look at one of them the person is not neat, there is rubbish at where she is cooking that is how you would know that the food you will be buying is not good and it will bring you diseases so it’s not good for you.’* (R15).

*‘It is not everywhere you have to eat. In some places when you get there and you want to eat, you have to look at the surroundings. Yes. But it is not everywhere you have to eat may be when you get to a place and you want to eat, you might not know the place and even if you know how the place is, you should be able to know whether the food they cook to sell is good or not. When you know how hygienic their food is then you can buy and eat from that place.’* (R19).

### Beliefs of healthy and unhealthy eating habits

Eating was considered an activity that structures daily life and maintains the human body. Healthy eating was perceived among the participants as a means to care for the body. This required a personal examination of any meal before consumption. According to some respondents, individuals who cared for their body did not eat harmful foods or ‘*anything, just like that*’ (R3 and R8) and also consumed plenty of water. Healthy eating was described by participants in three ways: timing of meals, portion control and food diversity.

#### Timing of meals

Healthy timing of meals was summarised by a participant as eating between 6 am to 6 pm. According to mothers, breakfast needs to be served before 10 am. Missing breakfast was seen as an unhealthy dietary practice. According to participants, healthy timing of meals also involved not eating continuously and allowing time for digestion before the next meal was taken. Participants reported that avoiding late meals enabled sleep, prevented diseases and facilitated digestion.

*‘The person controls his/her diet very well. The person does not eat at any time, maybe in the morning the person will eat rice, in the afternoon the person will eat gari [dried grit cassava] and sugar then in the evening the person will eat small.’* (R1)

‘*Yeah, let’s say I for one I don’t normally eat after six in the evening, because if I do, I can’t sleep, so that maybe what I will eat, normally at five let’s say I will take in fufu, after five I don’t take in anything at all, I don’t take in anything again so that it will digest if I eat after 6 pm it won’t digest well for me*.’ (R6).

More than half of the participants considered eating after 6 pm and especially at 10 pm as unhealthy (*n* = 17). According to them, eating late was unhealthy because one could not walk about after eating at such hours (*n* = 5).

#### Portion control: Not eating too much/ too much of everything is bad

Although we did not explore ideas about food portions using standard measures during the interview, eleven participants described healthy eating as not eating very large amounts of food. Overeating was considered a sin, a behaviour that frustrates the body and an act that can be avoided if individuals are conscious of it. Three participants narrated the challenges of over-eating as follows:

*‘It is not good because in this world our elders have made us to know that too much of everything is bad, so whatever you do in this world you have to be mindful of it and consider if it is the right thing that you are doing. Hmm like food that you have been asked to eat one-and-a-half balls of kenkey then you force yourself to eat two, it is a sin unto you.’* (R8)

*‘When you eat too much it makes the body frustrated [laughs] it does not give the body freedom, when you eat too much and let’s say you are going to check on something you cannot go with your friends you are tired and can’t even sleep, you have overeaten that is not good.’* (R15)

*‘It is not good because eating too much food at a time is not good. So if you eat a little, then in the afternoon you can also eat the rest’* (R6).

#### Eating different foods

Healthy eating was perceived as eating a variety of meals. Healthy eating was perceived as not eating the same meal twice a day and not more than thrice a week. Individuals perceived healthy eating as consuming different meals in a day; consuming the same meal for breakfast, lunch and supper was unhealthy. The consumption of a meal several times daily or weekly was associated with emotional dissatisfaction. This practice was described as a bad behaviour associated with food boredom and tiredness (“*afono* me” in Twi language R10) as explained below:

*‘You don’t have to eat kenkey [cooked fermented corn dough] every day, you don’t have to eat banku every day, and you don’t have to drink tea [beverage] every day. At times you can eat rice, you can eat yam, eh plantain in order to get carbohydrate. If every day you eat, eh, you eat carbohydrate foods you have to change it for your body to get strength. So we have to eat foods full of iron and calcium. That is why we alternate the foods we eat. One way eating, it is bad and you will not feel, you will not feel enjoy the place you are, me I felt tired*.*’* (R10)

In addition to eating a variety of foods, healthy eating was also perceived as choosing time appropriate meals. Even though participants advocated for eating a variety of foods, healthy meal selection was to be informed by time. In particular, *‘heavy meals’* [high energy foods] such as *kenkey, banku and fufu* were not to be consumed at night, even though consuming such might help a person to achieve food diversity.

### Factors that influence food choice

Participants mentioned four factors that influence their food consumption behaviour: finance, physiological and psychological factors, significant others and community.

#### Finance

More than half of the participants considered their finances when selecting food. When buying food from vendors, the income of the participants informed whether they bought nourishing foods (such as those served with soup) or those with limited nutrients (such as foods served with grounded pepper).

*‘The day I decide to eat fufu then there is money on me, then I decide to eat fufu, but if there is no money I have to buy kenkey’* (R11).

When participants were preparing meals home, the money available to them and the proportion they wanted to reserve for bills, school fees and other expenses informed the ingredients they used for cooking. Sometimes they left out some ingredients (*n* = 2), used small portions of some ingredients and/or bought less expensive versions of the ingredients (*n* = 3).

*‘Any food eaten by a rich person I could prepare it and eat it. When I feel like eating it and I have even GHS10.00 (USD$5.2)*^1^ *I can prepare it in my way, that same meal a rich person will use GHS100.00, I will use GHS10.00 to prepare. Maybe the rich person will buy tomatoes GHS10.00, I will buy tomatoes GHS2.00, and then I will buy onion GHS0.50 and use it. Every ingredient in small quantities [bibia kakrakakra,* in Twi language*]. But for the rich man they will buy in bulk and cook it big, but for me if I have small money I can use it to prepare it.’* (R3).

From the account of two of the respondents when there was no money to make a meal, it was better to use a few ingredients to make “*dry”* versions of the food. Foods such as *oiled rice*, and dry jollof (jollof rice made with fewer vegetables and no fish/meat) were such alternatives.

*‘You can make some dry jollof.... You make it with little tomatoes, one tin, with a small saucepan, then you eat with your kids’* (R7).

*‘Yesterday, we ate some jollof in the morning [...]. Luckily I had GHS 2.00, I just gave it to her [his sister], we were having some rice given to me by a friend then my second son brought some from his office and gave me one bag [5kg of rice], so I gave her one rice with GHS2.00, she went to the market got some tomatoes without meat, oh, without fish oh before God and man, all of us we ate we were satisfied, I, my grandchildren, even my daughter came and had the under [the burnt portion of the rice].’* (R5).

In addition, to reduce food expenditure some participants avoided foods that do not produce and sustain feelings of satiety, foods that were served in small portions and those that could not feed the entire family. These were mainly fruits and vegetables.

*‘When I buy it* [fruits]*, is good. But to get it, the thing, that is where the problem lies [na ei bi wahala]. Fruits are good or fruits is good, so when I say no there is pineapple, apple and etc. Let me go and take some, and I buy that thing, maybe sister* [*ekom be we me*] *I will starve [laughs]. (Interviewer and respondent laughs) Oh true fact [laughs] true, you see, oh, let me speak the truth, let me speak the truth [ekom be we me] I will starve, you see.’* (R10).

*‘Yea, it is expensive because if I eat not I alone, I must eat my wife, my children, assuming my daily mark is GHS5.00, so in the morning we use that alone as breakfast how much will remain for the afternoon and evening, and how much will remain for my electricity bill and water bill. The most expensive is the school fees.’* (R5).

#### Physiological and cognition/emotional factors

According to the participants their bodily and mental status also informed their food decisions. Ten participants mentioned that their mind tells them what to eat, *‘what the mind tell me,*’ and for eight it was what they ‘*feel for*.’ Among four of the participants, food choice was informed by their energy needs, whether their body needed to replenish lost energy or to be pampered after a strenuous task. For two participants satiety was the first factor that informed their food selection.

In addition, food preference was mentioned by participants. For example some respondents did not like some fruits (such as strawberry, apples, pawpaw, and mango), meals made from corn, or meals with lots of pepper or high salt content. The following excerpt is an example:

*‘I don’t like it, apple like this I don’t like it no matter what, and I don’t eat it. Aha, and avocado pear I don’t go and buy anyhow, when I see it sliced and it happens to be the type I like aha I will eat, but for me to go and buy and it will be watery or something that one also I don’t like it. But what I truly like is the orange that is the only thing that I always eat.’* (R13)

#### Community

The community was also mentioned as a factor that influenced food choice. In particular, the availability and affordability of foods were mentioned. Four participants mentioned that fruits and vegetable stews were not available in their community. Fruits vendors were inconsistent with their trade. In addition, most food vendors did not sell vegetable-based sauces like *palava sauce*.

*‘This place too sometimes what I want to eat is not available then I take my mind off hunger.’* (R20).

The food vendors often did not sell vegetable salads and locally perceived healthy stews such as *palava sauce*. The most dominant vendor food is kenkey served with grounded pepper, fried fish and shito (the fish and *shito* are prepared with refined vegetable oil). Rice meals are the second dominant vendor food. The rice vendors sell polished white rice dishes served with tin tomato sauces, fried frozen chicken, fried fish, canned sardines and fried egg. Also, the convenient stores sell variety of processed foods, mostly sweets and alcohol. Two participants believed that food is less expensive in the community compared to other areas in Accra. A resident of Agbobloshie mentioned that vegetables and fruits are less expensive at the Agbobloshie markets.^2^

*‘Yes, over here [referring to Agbobloshie] we buy local foods when you go to the town you will not buy it at the same price like over here, over here we can buy rice at 50pesewa or 1cedis but when you go to the town like Osu and the rest you can't go and buy rice at 50pesewa.’* (R22).

*‘If you go to the market there, is there, is very very cheap, but am not buying it you see oh.’* (R10).

#### Significant others

Some participants mentioned the influence of their mothers (*n* = 6), wives (*n* = 3), siblings (n = 3), children (*n* = 2) and friends (n = 2) on their food choices. Mothers in particular were very influential in teaching their children how to cook and what foods to eat. As adults the socialisation they received from their mothers informed their food choice. Some participants had adopted the cooking style of their mothers. Among the men, the type and content of their food were dependent on their mothers, aunts, sisters and wives who did the cooking. Among peers, food recommendations were also suggested. These were usually processed foods such as savoury snacks, soda and instant noodles.

The size of the household informed the source of the meal. Two participants did not cook because they lived alone and had none to share a meal with. They resorted to street food vendors. In addition, in large size households priority was given to carbohydrates over vegetables and proteins during meal preparation.

## Discussion

This study focused on three empirical areas: (1) food beliefs, (2) beliefs of healthy and unhealthy food practices and (3) factors that influence food choice. The discussion is organised around these themes. We also consider the implications of the food beliefs and factors that influence food choice for CVD prevention and management.

### Food beliefs

Food was interpreted as edible natural resources that are socially acceptable and that aid physical growth. This definition reflects the idea that what is considered edible and a form of sustenance is a function of social and cultural meanings [55]. These resources/food in their fresh form were seen as good and healthy. This idea supports other studies that have reported that fresh foods are seen as healthy [52].

Foods that had been part of the Ghanaian traditional diet (e.g. fufu, ampesi, palm oil) were considered healthy compared to foreign foods (e.g. fried rice, instant noodles and processed food) and fatty foods [56]. Previous research in Ghana and dietary guidelines, reports that oily foods are unhealthy and associated with diabetes risk and prohibited for some pregnant women [55]. The findings suggest that while culture informs food beliefs, some beliefs are aligned with recommended dietary guidelines.

### Content of beliefs of healthy cooking and healthy eating practices

According to the participants, food becomes unhealthy to the body depending on how it is cooked and consumed. When discussing their understanding of healthy and unhealthy food practices, the focus of participants shifted from the cultural to the individual. Emphasis was placed on the body as the means of determining what is healthy or unhealthy. Healthy cooking habits involved selecting the best ingredients, using appropriate cooking methods, and maintaining food hygiene as has been found in other studies [53, 54].

Even though most Ghanaian stews involve frying, frying was considered an unhealthy cooking method because it could introduce excess oil into a meal if it is not done properly [40]. Maintaining food hygiene was mentioned as a healthy food practice for both health and social reasons. There is a growing consciousness in Ghana that food poisoning can result from contaminated food and individuals adopt risk avoidance strategies like assessing cleanliness before buying food from vendors [54]. Unhygienic meals were perceived as harmful to the body in line with expert dietary guidelines.

Healthy eating involved eating the right meals, in the right quantity, and at the right time. In agreement with the recommendation of the dietary guidelines for Ghana, eating after 6 pm was considered as unhealthy by participants [29]. In addition, late meals have also been associated with diabetes in Ga Mashie [25].

Participants believed that achieving food diversity is a healthy eating habit; however, their understanding of food diversity was restricted to consuming different meals instead of different food groups as experts recommended [57]. This misunderstanding in knowledge can negatively affect their diet quality. Although participants considered overeating as unhealthy, this idea was not linked to considerations of food portions.

### Multi-level factors that influence food choice

The participants mentioned four factors that influenced their food choice; physical and psychological state, significant others, community and finances. These factors were aligned with the different levels proposed in the socio-ecological model. At the intrapersonal level, the body; mind, feelings and embodied nutritional needs were mentioned as influencing food choice. This finding is aligned with discussions on the impact of food preferences and psychological processes on food habits [58, 59].

In Ghana, previous studies have shown the influence of inter-personal relations on food habits. For example, mothers/aunts enforce breastfeeding norms on their daughters [55] and women are the cooks and those who teach others how to cook [40]. In this study, we found the influence of mothers, wives, siblings and peers shaping the food choice of participants.

The community influenced participants’ access to healthy foods. In Ga Mashie, there is a proliferation of street food vendors and convenience stores, however unhealthy foods outnumber healthy options. The findings support research using global positioning systems in Ga Mashie has concluded that there is a food dessert: fruits and other fresh foods are not available in the community [13].

In this study, the lack of money limited healthy food practices. The structures of urban areas mean that people rely on the market for their food supply and most food commodities are purchased [34]. This makes money a key component of the urban food market. Urban poor residents usually have limited cash and cash reserves and are more vulnerable to price changes [60].

## Conclusions

This study examines lay perspectives of food and healthy eating, drawing on interviews with urban poor residents in Accra, Ghana. From a conceptual viewpoint, this study is aligned with current debates about the interplay between individual and environmental influences on health-related behaviours [34, 35, 37, 61, 62]. From a policy viewpoint, this research is aligned with global and national policy emphasis on promotion of healthy eating and lifestyles in communities through nutrition education [20, 38, 39]. Ghana’s RHN programme is consistent with the global public health policies aimed at addressing the risk and burden of NCDs [63, 64].

Three findings of this study are relevant to the RHN campaign. Firstly, this study shows that natural foods are considered healthy. This insight is vital for encouraging the consumption of fresh foods such as fruits and vegetables while limiting consumption of ultra-processed and highly processed foods. The consumption of fresh foods is advocated for addressing obesity, hypertension, diabetes and related complications [16, 18, 63].

Secondly, this study found that lay people are knowledgeable of the influences of cooking and eating practices on health. This idea can be incorporated into health promotion since people are far more likely to change their behaviour if the message is built on existing lay knowledge [30, 65]. For example, the belief in “not (eating) too much” promotes eating in moderation and indicates the desire to avoid excessive eating. Community health workers can provide a visual illustration of the portion and serving sizes of food items and meals.

A third insight is that lay people perceive healthy foods as expensive. In similar studies, the cost of food was reported as a barrier to adopting healthy 683 eating [12, 20]. The cost of food and poverty operates at the macro level [41]. In general, deprived neighbourhoods report unhealthy dietary patterns. In the RHN programme and policy, the Ministry of Health committed to collaborating with other government agencies such as local government, sanitation and education to create and support healthy communities and homes [38, 39]. Delivering on this promise and committing to regulate market forces that influence food price can greatly affect dietary change.

This study has some limitations. The discussion on food quantity could have been improved by probing on food portions with standard visual measures. We did not observe everyday food practices. Future studies can focus on objective measures of food portions as well as explore ideas on food diversity. However, the study identified the multilevel dimensions of food beliefs and practices, which offer clear, specific insights for developing culturally appropriate and context-specific interventions.

## Additional files

Additional file 1: Detailed background characteristics of study respondents. Description of data: this file provides detail information about the socio-demographic charcteristics of the study participants (DOCX 17 kb)

Additional file 2: Description of food items. Description of data: this file provides description of the food items listed in the study. (DOCX 11 kb)

## Footnotes

1 The exchange rate at the time of data collection was USD$1 to GHS1.9

2 Agbobloshie is a popular market for wholesale purchases of staple foods like fruit, vegetables and tubers for households of all income levels in Accra
