# Supplementary material for: Food beliefs and practices in urban poor communities in Accra: implications for health interventions
Source: BMC Public Health. 2018 Apr 2;18:434. doi: 10.1186/s12889-018-5336-6 (PMC5880073; doi:10.1186/s12889-018-5336-6)
Supplement: Supplementary file 1 — Detailed background characteristics of study respondents. Description of data: this file provides detail information about the socio-demographic charcteristics of the study participants (DOCX 17 kb) [file 12889_2018_5336_MOESM1_ESM.docx]

**Additional File**

**Food beliefs and practices in urban poor communities in Accra: implications for health interventions**

Boatemaa S, Badasu, D. M. and de-Graft Aikins, A.

**Additional file 1: Detailed background characteristics of study respondents**

| **Appendix 1. Detail characteristics of respondents, qualitative data^[[1]](#footnote-1)^** | | | | | | | |
| --- | --- | --- | --- | --- | --- | --- | --- |
| **Variable** | | | | | | | |
|  | **Sex** | **Marital status** | **Locality** | **Ethnicity** | **Education** | **Religion** | **History of NCD** |
| R1 | M | Never married | Agbogloshie | Ga-Dangme | Secondary | Christian | Nil |
| R2 | M | Married | Agbogloshie | Akan | Middle/JSS | Christian | Nil |
| R3 | F | Divorced | Agbogloshie | Akan | Middle/JSS | Christian | Nil |
| R4 | F | Divorced | Agbogloshie | Akan | Primary | Christian | Nil |
| R5 | M | Never married | Agbogloshie | Akan | Secondary | Christian | Nil |
| R6 | F | Married | Agbogloshie | Akan | Middle/JSS | Christian | Nil |
| R7 | F | Married | Agbogloshie | Akan | None | Christian | Nil |
| R8 | F | Never married | Agbogloshie | Mole-Dagbani | Higher | Muslim | Nil |
| R9 | M | Never married | James Town | Ewe | Secondary | Christian | Nil |
| R10 | F | Divorced | James Town | Ga-Dangme | Primary | Christian | Nil |
| R11 | F | Married | James Town | Ga-Dangme | Primary | Christian | Nil |
| R12 | M | Never married | James Town | Ga-Dangme | Middle/JSS | Christian | Nil |
| R13 | F | Never married | James Town | Ga-Dangme | Middle/JSS | Christian | Nil |
| R14 | M | Never married | James Town | Akan | Secondary | Christian | Nil |
| R15 | M | Never married | James Town | Ga-Dangme | Secondary | Christian | Nil |
| R16 | M | Married | James Town | Ga-Dangme | Secondary | Christian | Nil |
| R17 | F | Never married | James Town | Akan | Middle/JSS | Christian | Nil |
| R18 | F | Divorced | James Town | Ga-Dangme | Primary | Christian | Nil |
| R19 | F | Widowed | James Town | Ga-Dangme | None | Christian | Nil |
| R20 | F | Married | James Town | Ga-Dangme | None | Christian | Nil |
| R21 | M | Never married | James Town | Ga-Dangme | Secondary | Christian | Nil |
| R22 | M | Never married | Ussher Town | Ga-Dangme | Middle/JSS | Christian | Nil |
| R23 | F | Married | Ussher Town | Ga-Dangme | Tertiary | Christian | Nil |
| R24 | F | Divorced | Ussher Town | Ga-Dangme | Middle/JSS | Christian | Nil |
| R25 | F | Divorced | Ussher Town | Ga-Dangme | Middle/JSS | Christian | HBP |
| R26 | F | Never married | Ussher Town | Ga-Dangme | Middle/JSS | Christian | Nil |
| R27 | M | Married | Ussher Town | Ga-Dangme | Secondary | Christian | Nil |
| R28 | M | Married | Ussher Town | Akan | Secondary | Christian | Nil |
| R29 | M | Married | Ussher Town | Ga-Dangme | Secondary | Christian | Nil |
| R30 | F | Divorced | Ussher Town | Ga-Dangme | Primary | Muslim | Nil |

1. The ages of the participants has been removed to ensure their anonymity [↑](#footnote-ref-1)
