# Supplementary material for: Food beliefs and practices in urban poor communities in Accra: implications for health interventions
Source: BMC Public Health. 2018 Apr 2;18:434. doi: 10.1186/s12889-018-5336-6 (PMC5880073; doi:10.1186/s12889-018-5336-6)
Supplement: Supplementary file 2 — Description of food items. Description of data: this file provides description of the food items listed in the study. (DOCX 11 kb) [file 12889_2018_5336_MOESM2_ESM.docx]

**Additional File**

**Food beliefs and practices in urban poor communities in Accra: implications for health interventions**

Boatemaa S, Badasu, D. M. and de-Graft Aikins, A.

**Additional file 2: Description of food items**

| **Appendix 2. Description of food items** | |
| --- | --- |
| **Food item** | **Description** |
| Akorlor | Cooked seasoned fresh fish |
| Banku | Fermented corn meal pudding |
| Fufu | Pounded boiled cassava with plantain or cocoyam |
| Gari | Dried grit cassava |
| Kelewele | Fried spicy ripe plantain cubes |
| Kokonte | Cassava flour pudding |
| Koose | Fried bean cake |
| Shito | Pepper sauce |
| Palava sauce | Cocoyam leaves stew with melon seeds and fish |
| Tatale | Fried ripe plantain fritters |
| Togbee | Sugar doughnut |
